# Assessment of the Effectiveness of a Visual Coaching Device Combined with Auditory Instructions on Reproducibility and Stability in Deep Inspiration Breath-Hold Radiotherapy

**DOI:** 10.3390/jcm14207259

**Published:** 2025-10-14

**Authors:** Yoon Young Jo, Eunseo Bae, Ji Woon Yea, Jae Won Park, Jaehyeon Park, Se An Oh, Hyunyeol Lee

**Affiliations:** 1 Department of Radiation Oncology, Yeungnam University Medical Center, Daegu 42415, Republic of Korea; cantabile801@naver.com (Y.Y.J.);; 2 School of Electronic and Electrical Engineering, Kyungpook National University, Daegu 41566, Republic of Korea

**Keywords:** deep inspiration breath-hold, breast cancer, visual-auditory guidance, reproducibility, stability

## Abstract

**Background/Objectives**: In left-sided breast cancer radiotherapy, deep inspiration breath-hold (DIBH) reduces radiation dose to the heart; however, its success depends on the patient’s ability to maintain reproducible and stable breath-holds. Although visual and auditory guidance systems have been introduced to improve performance, quantitative data on their combined effects remain limited. **Methods**: We retrospectively analyzed 400 breath-hold sessions in 20 patients undergoing DIBH treatment using synchronized visual and auditory coaching. Real-time respiratory displacement data were used to calculate reproducibility and stability. Temporal trends and correlations between reproducibility and stability were also assessed. **Results**: The mean reproducibility was 0.51 ± 0.22 mm (range, 0.20–1.10 mm), and the mean stability was 0.77 ± 0.21 mm (range, 0.50–1.16 mm). Both measures showed significant improvement over the course of treatment, indicating a learning effect. Linear regression analysis revealed a significant positive correlation between poorer reproducibility and greater intrafractional instability (R^2^ = 0.26, *p* = 0.021). Daily cone beam computed tomography-based image-guided radiotherapy demonstrated minimal isocenter shifts, confirming consistent patient setup with DIBH. **Conclusions**: Combined visual and auditory coaching during DIBH yielded low variability in reproducibility and stability, with progressive improvements over time. These findings highlight the ability of multimodal feedback systems to enhance respiratory consistency and support their integration into clinical practice, optimizing treatment precision.

## 1. Introduction

Breast cancer remains one of the most common malignancies among women worldwide. Radiotherapy plays a crucial role in reducing local recurrence and improving survival rates; however, unintentional radiation exposure to the heart, particularly the left anterior descending (LAD) coronary artery, poses a significant long-term risk of radiation-induced heart disease (RIHD), particularly in patients with left-sided breast cancer [1,2,3,4]. 

A landmark study by Darby et al. demonstrated a linear relationship between the mean heart dose and the risk of major coronary events, with a 7.4% increase in risk per 1 Gy of heart exposure [5]. Therefore, techniques that minimize cardiac dose are essential for treating left-sided breast cancer.

Deep inspiration breath-hold (DIBH) is a widely adopted method for cardiac sparing that increases thoracic volume and displaces the heart away from the chest wall, reducing cardiac dose [6]. DIBH can significantly reduce the dose to the heart and lungs without compromising target coverage [7]. Multiple studies have confirmed that DIBH significantly reduces radiation exposure to cardiac structures [8,9]. A recent meta-analysis by Lu et al. [10] demonstrated mean heart dose reductions ranging from 26% to 75% with DIBH compared to those with free breathing. In addition, Falco et al. [11] highlighted the consistent heart-sparing benefits of DIBH across various breathing control techniques. Despite its clinical benefits, the effectiveness of DIBH largely depends on the patient’s ability to perform and maintain a consistent breath-hold throughout treatment [12].

The two key metrics used to assess the quality of DIBH are reproducibility (RPD), which refers to the ability to return to the same breath-hold position across sessions, and stability (STB), which refers to the ability to maintain a steady position during a single breath-hold. Inconsistencies can lead to geometric uncertainties or compromised treatment precision. Various patient guidance systems have been introduced to enhance DIBH performance. Visual coaching, which utilizes display monitors, and auditory feedback, such as voice cues or beeping sounds, helps patients achieve consistent breath-hold levels [7,13]. However, despite the increasing use of multimodal guidance, quantitative data validating its effectiveness in improving RPD and STB are limited.

In this study, we evaluated the breath-holding performance of 20 patients with left-sided breast cancer who underwent DIBH under visual and auditory guidance. By analyzing session-wise breath-hold data during treatment, we sought to quantify RPD and STB, assess temporal trends, and determine the impact of the multimodal guidance system. We hypothesized that synchronized audiovisual coaching would help patients achieve reproducible and stable breath-holds during radiotherapy.

## 2. Materials and Methods

**Figure 1:**
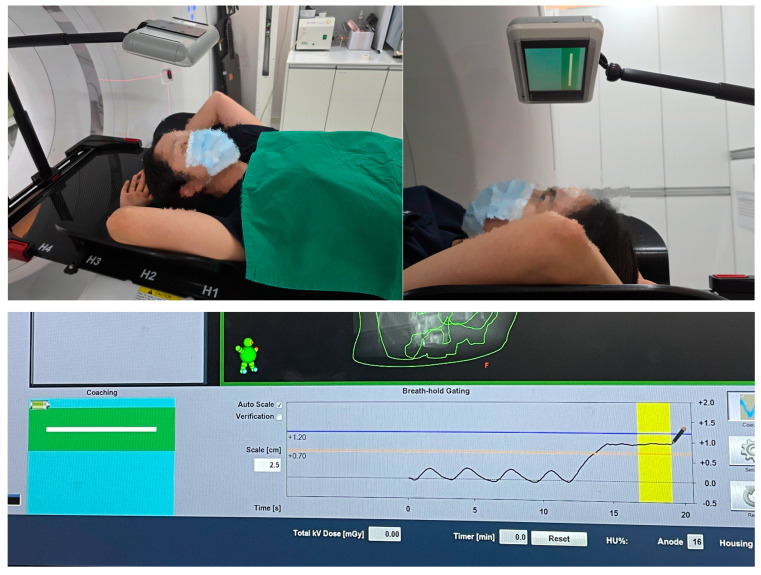
Setup for deep inspiration breath-hold (DIBH) using synchronized audio and visual coaching. (**Top**) A patient performs DIBH while receiving visual feedback through a monitor and auditory instructions from the operator. (**Bottom**) Real-time respiratory monitoring system displaying the breath-hold guidance interface and displacement trace.

Between January 2024 and March 2024, 20 patients with left-sided breast cancer who received radiotherapy using the DIBH technique at Yeungnam University Medical Center were recruited. Inclusion criteria encompassed patients with pathologic stage I–IIa breast cancer who underwent breast-conserving surgery and were able to perform three consecutive, stable 15–20 s DIBHs at simulation with audiovisual guidance. Exclusion criteria were patients unable to meet the above requirement. The median age of the patients was 52 years (range, 42–74). Radiotherapy was delivered using IMRT with a simultaneous integrated boost technique at a dose of 5200/4800 cGy in 20 fractions. Target contours were delineated according to the institutional protocol for whole-breast irradiation. All patients were guided during DIBH using a combination of visual coaching (Varian RGSC system, version 2.0, Varian Medical Systems, Palo Alto, CA, USA, with monitor display) and auditory cues (in-house tablet-based voice instruction system) to maintain a consistent breath-hold level (Figure 1). Each patient completed 20 treatment fractions, resulting in 400 breath-holding sessions for analysis.

### 2.1. Data Acquisition and Processing

Real-time respiratory signals were recorded using an in-room monitoring system capable of measuring vertical displacement during breath-holding. RPD and STB are expressed in millimeters, corresponding to the units used for the patient setup. For a series of DIBHs, RPD was defined as the maximum difference between the different DIBH levels; the more varied the DIBH levels, the larger the value. STB was defined as the maximum amplitude change between the initial and end time points of a DIBH when fitted by a least-squares line. The data were extracted in a time-series format and processed using custom Python-based scripts to calculate breath-hold performance metrics.

### 2.2. Evaluation Metrics

**Figure 2:**
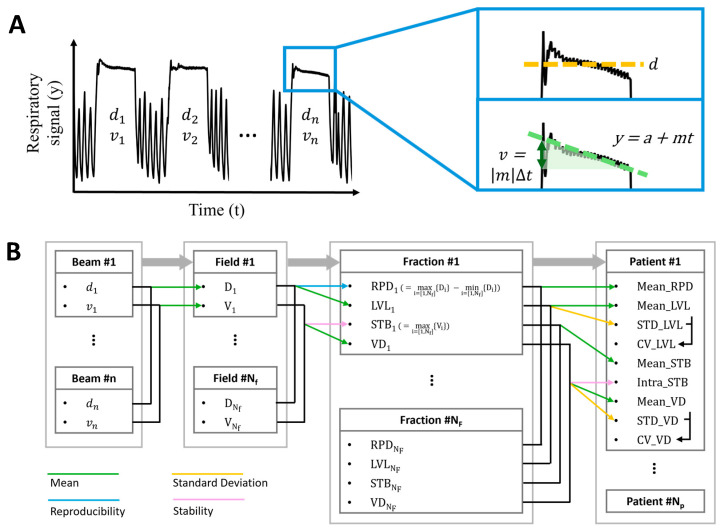
Estimation of patient breath-hold performance from respiratory signals across beam (**A**), field, and fraction levels, yielding nine evaluation scores for each patient (**B**).

To evaluate patient compliance with breath-holding instructions during radiation therapy, the following metrics were assessed: averaging RPD (Mean_RPD), averaging LVL (Mean_LVL), standard deviations of LVL across all fractions (STD_LVL), coefficients of variation in LVL (CV_LVL), averaging STB (Mean_STB), intra-patient stability representing patient’s overall breath-hold performance for the entire therapeutic period (Intra_STB), averaging VD (Mean_VD), standard deviations of VD across all fractions (STD_VD), and coefficients of variation in VD (CV_VD). To derive these scores, two base values, in millimeters, were first computed from the records of the patient’s abdominal positions during each breath-hold time (Figure 2A): mean level (d) and end-to-end deviation (v), estimated using linear regression as follows:

(1) $$v=\left|m\right|∙∆t$$

where m is the slope of the regression line, and ∆t is the duration of the breath-hold.

Figure 2B shows the stepwise process used to derive metrics. Averages of d and v across the breath-holds were calculated, resulting in D and V values for each field of radiation therapy. RPD and STB for each fraction were computed using the following equations [13]:

(2) $$RPD=\underset{j=[1,N_{f}]}{\max}\left\{D_{j}\right\}-\underset{j=[1,N_{f}]}{\min}\left\{D_{j}\right\}$$

(3) $$STB=\underset{j=[1,N_{f}]}{\max}\left\{V_{j}\right\}$$

where N_f_ is the number of fields per fraction, and RPD and STB represent the fraction-level consistency of breath-holds. Lower values indicate better performance.

In addition, the averages of D and V across the fields were calculated, yielding LVL and VD, respectively, for each fraction. Finally, the nine measures were derived for each patient. Mean_RPD, Mean_LVL, Mean_STB, and Mean_VD were obtained by averaging RPD, LVL, STB, and VD, respectively, whereas STD_LVL and STD_VD were obtained from the standard deviations of LVL and VD across all fractions. CV_LVL and CV_VD were calculated by taking the ratio of the standard deviations to the averages of LVL and VD. Furthermore, the intrapatient STB (Intra-STB), representing the patient’s overall breath-hold performance across the entire therapeutic period, was derived as follows:

(4) $$Intra\_STB=\underset{k=[1,N_{F}]}{\max}\left\{{VD}_{k}\right\}$$

where N_F_ is the number of fractions.

### 2.3. Statistical Analysis

Descriptive statistics (mean, standard deviation, and range) were calculated for all parameters. Linear regression was used to evaluate temporal trends across treatment fractions and to assess the relationship between RPD and STB. Statistical significance was set at *p* < 0.05. All analyses were performed using Python (v3.11). 

## 3. Results

### 3.1. Overview of RPD and STB Evaluation

Table S1 summarizes the nine performance evaluation scores (Mean_RPD, Mean_LVL, STD_LVL, CV_LVL, Mean_STB, Intra_STB, Mean_VD, STD_VD, and CV_VD) obtained for all 20 patients, along with their group averages.

### 3.2. RPD of Breath-Hold Level

**Figure 3:**
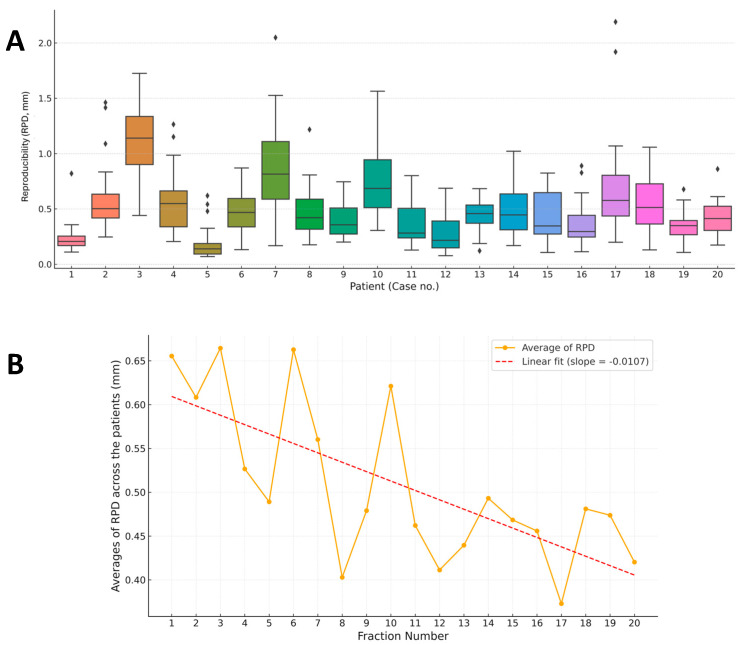
Reproducibility of breath-hold performance. (**A**) Box plots of patient-specific reproducibility (RPD) across all 20 patients. (**B**) Temporal trend of average RPD, with a linear regression line indicating gradual improvement over successive fractions.

The patient-wise distribution of RPD is shown in Figure 3A. Most patients maintained a consistent breath-hold height with minor RPD variability. Across the 20 patients, the mean RPD of breath-hold level was 0.51 ± 0.22 mm (0.20–1.1 mm). However, several patients demonstrated broader interquartile ranges and outliers, indicating fluctuations in breath-hold control.

Linear trend analysis revealed that average RPD improved over time, accompanied by a steady decrease in variation across treatment sessions (Figure 3B). This suggests that patient adaptation and increased consistency can be achieved through repeated DIBH training under multimodal guidance. Repeated-measures analysis of variance confirmed significant improvements in RPD over time (RPD: F(19,361) = 2.41, *p* = 0.0009).

### 3.3. STB of Breath-Hold Across Fractions

**Figure 4:**
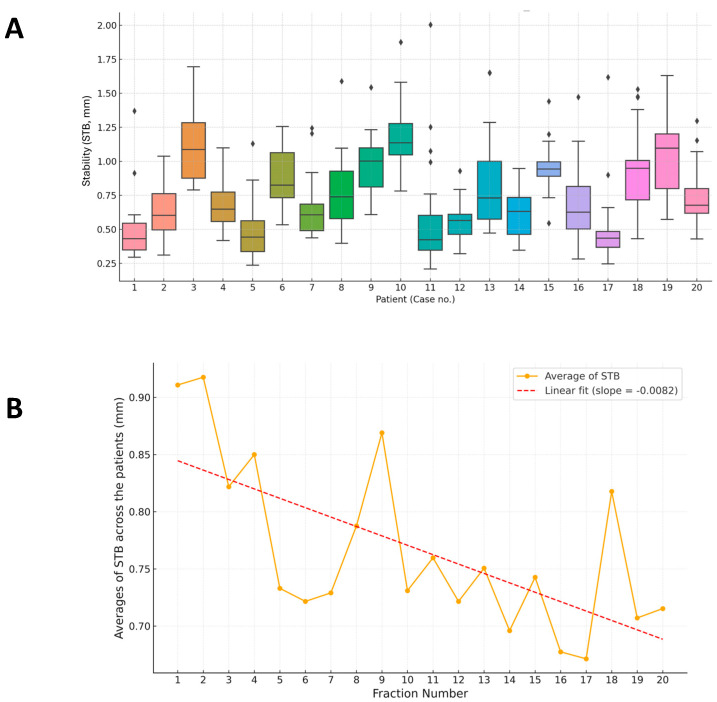
Breath-hold stability analysis. (**A**) Box plots of patient-specific stability (STB) across all 20 patients. (**B**) Temporal trend of average STB with a linear regression line, demonstrating progressive improvement across treatment fractions.

Figure 4A illustrates the STB distribution. Although most patients exhibited tight and consistent distributions, the mean interfraction STB (Mean_STB) across patients was 0.77 ± 0.21 mm (0.50–1.16 mm). Certain individuals demonstrated greater variability, indicating challenges in maintaining a stable breath-hold positioning across sessions.

Linear trend analysis of STB (Figure 4B) revealed a downward trend, confirming that STB progressively improved over time, consistent with increased patient familiarity and adherence to the protocol. Repeated-measures ANOVA confirmed significant improvements in STB across treatment fractions (STB: F(19,361) = 1.64, *p* = 0.044).

**Figure 5:**
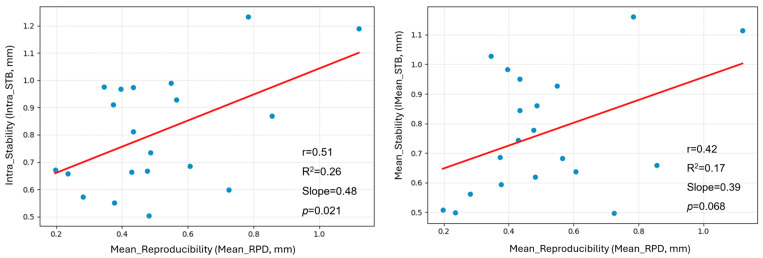
Scatter plots illustrating the relationship between breath-hold reproducibility (Mean_RPD) and stability parameters. (**Left**) A significant positive correlation was observed between Mean_RPD and Intra_STB. (**Right**) Mean_RPD also showed a positive trend with Mean_STB.

To evaluate the relationship between breath-hold RPD and STB, linear regression was performed (Figure 5). A significant positive correlation was observed between Mean_RPD and intrafraction STB (Intra_STB) (r = 0.51, R^2^ = 0.26; *p* = 0.021), suggesting that patients with poor RPD tend to exhibit reduced STB within breath-hold sessions. In contrast, Mean_RPD showed a borderline correlation with Mean_STB (r = 0.42, R^2^ = 0.17; *p* = 0.068), indicating a possible trend toward reduced interfractional consistency in patients with less reproducible breath-holds. After applying Bonferroni correction for two prespecified comparisons, the association between Mean_RPD and Intra_STB remained significant (adjusted *p* = 0.042), whereas the correlation between Mean_RPD and Mean_STB did not (adjusted *p* = 0.136).

### 3.4. Case-Specific Analysis of Breath-Hold Level

**Figure 6:**
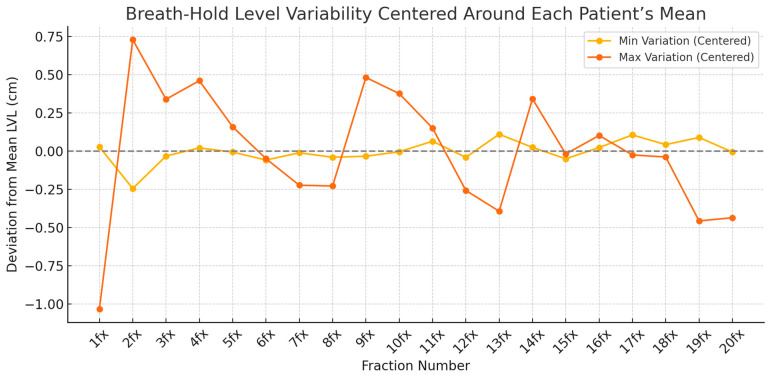
Comparison of breath-hold level variability between two representative patients. The yellow line indicates the patient with the smallest deviations from the mean level across fractions, whereas the orange line represents the patient with the largest deviations.

Figure 6 compares breath-hold amplitudes between patients with the least and most variation. Patient 5 exhibited highly stable breath-hold levels with minimal deviation, whereas Patient 10 showed large fluctuations over the course of treatment, highlighting interindividual differences in breath-hold control.

### 3.5. Image-Guided Radiotherapy Results of All Treatment Sessions

Daily image-guided radiotherapy using cone beam computed tomography (CT) was performed for all treatment fractions. The mean isocenter shifts in the vertical, longitudinal, lateral, pitch, roll, and rotation directions were minimal, with median values (standard deviation) of −0.23 cm (0.23), 0.06 cm (0.37), −0.03 cm (0.15), 0.15° (1.08), 0.05° (0.56), and 0.00° (0.58), respectively (Table S2). These results indicate that patient setup variations were small and that using DIBH during radiotherapy maintained isocenter STB throughout treatment.

## 4. Discussion

We retrospectively analyzed 20 patients with left-sided breast cancer undergoing radiotherapy to quantitatively evaluate the impact of combined visual coaching and auditory instruction on DIBH performance. RPD was defined as the maximum difference in the average breath-hold level across 20 treatment fractions, whereas STB was defined as the maximum amplitude change between the initial and end points of each DIBH session. Based on real-time respiratory signal data from 400 breath-hold sessions, we found that most patients maintained highly consistent DIBH performance, with a mean RPD of 0.51 mm. RPD and STB improved progressively over the course of treatment, and a significant correlation was observed between poor RPD and reduced intrafraction STB.

Previous studies have demonstrated the beneficial effects of visual feedback (VF) systems in enhancing DIBH RPD [13,14,15,16,17]. For instance, Yamauchi et al. [16] reported that patients using VF exhibited significantly smaller chest-wall displacement (0.59 ± 3.64 mm) compared to that with auditory-only coaching (2.09 ± 4.96 mm; *p* < 0.001). Similarly, Hoshina et al. [14] utilized a laser-based sensor with VF to measure the chest wall–heart distance during DIBH and found interquartile range values approximately two to three times smaller than those without VF, indicating markedly improved interfraction RPD. Moreover, early implementation of surface imaging systems combined with visual coaching, as described by Cervino et al. [13], demonstrated improvements in RPD and STB during patient setup.

Several studies have also investigated the role of audio-based coaching in improving respiratory control during radiotherapy [18,19,20,21,22,23]. George et al. [18] demonstrated that audio-only instruction improved RPD compared to that with free breathing, although audiovisual feedback achieved superior control. Similarly, Sano et al. [22] compared visual and auditory guidance in patients with thoracic and abdominal tumors and found both approaches equally effective, with most patients preferring auditory cues because of their comfort and ease of use. Yu et al. [23] confirmed that audio-only feedback provides respiratory regularity comparable to audiovisual methods in cases of abdominal tumors. However, Nangia et al. [21] reported that, although audio-only coaching effectively regulated breathing frequency, it was less reliable in reproducing the amplitude of deep inspiration during DIBH for breast cancer. Collectively, these findings suggest that auditory guidance is a practical and patient-friendly strategy, but its effectiveness may vary depending on tumor site and is best complemented with VF to maximize breath-hold performance. Table S3 summarizes representative studies on DIBH with visual and/or auditory guidance.

Regardless of the specific feedback modality, progressive improvements in RPD and STB over the course of treatment suggested a clear learning effect. In our study, patients did not receive structured pre-training before radiotherapy. Therefore, the observed improvements likely reflect learning acquired during treatment fractions. Based on this finding, we speculate that introducing a structured pre-training session at CT simulation could enable patients to achieve stable and reproducible DIBHs from the first treatment fraction. This highlights the potential value of structured patient education and practice before treatment initiation. Supporting this, Kefeli et al. [24] demonstrated that patients who received dedicated coaching and instructional materials prior to CT simulation exhibited significantly shorter setup durations and lower LAD maximum doses during radiotherapy than did those who received standard technician-led instruction only on the day of simulation (29.5 Gy vs. 36.5 Gy, *p* = 0.02). These results indicate that in-room feedback systems and pretreatment preparation play complementary roles in optimizing breath-hold performance and, ultimately, clinical outcomes.

This study has some limitations. First, its retrospective design limits causal inference and introduces potential selection bias. Second, the small sample size may reduce generalizability despite the large number of breath-hold sessions analyzed. Third, the absence of a control group without audiovisual guidance prevents definitive attribution of performance improvements to the coaching system. Additionally, breath-hold quality was assessed using external surface displacement, which may not accurately reflect internal organ motion. Furthermore, no dosimetric data were analyzed, and variability in individual coaching experience was not controlled, which may have influenced the RPD and STB outcomes. Finally, this study was conducted exclusively in patients with early-stage breast cancer. Therefore, the generalizability of our findings to advanced-stage breast cancer involving regional nodal irradiation, or to other thoracic and abdominal tumors, remains uncertain. Further studies are warranted to validate the applicability of combined audiovisual coaching in these broader clinical contexts.

Although our study quantitatively assessed the effect of combined visual and auditory coaching on DIBH performance, future research should integrate real-time surface-guided radiation therapy (SGRT) systems to enhance motion management and setup accuracy. The large-scale SAVE-HEART study [25] demonstrated significant dosimetric advantages of surface-based DIBH with audiovisual feedback, including marked reductions in cardiac and pulmonary doses and projected cardiovascular risk. However, that study did not directly quantify intra- and interfractional breath-hold RPD using SGRT data. Future investigations combining quantitative breath-hold metrics with SGRT-based motion tracking are warranted to optimize patient selection, refine coaching protocols, and minimize geometric uncertainties during DIBH radiotherapy.

## 5. Conclusions

Although our study did not include single-modality control groups, our findings demonstrate that synchronized visual and auditory guidance in DIBH improves breath-hold RPD and STB over time, despite individual variations. Overall, multimodal coaching systems appear effective in enhancing respiratory consistency during radiotherapy.

## Data Availability

The data presented in this study are available on request from the corresponding author.

## Supplementary Materials

The following supporting information can be downloaded at: https://www.mdpi.com/article/10.3390/jcm14207259/s1, Table S1: Reproducibility and stability results of 20 patients across 400 treatment sessions; Table S2: Mean isocenter shift with daily image-guided radiation therapy; Table S3: Representative studies on DIBH using visual and/or auditory guidance.

## Abbreviations

The following abbreviations are used in this manuscript:

CT	Computed tomography
CV	Coefficients of variation
DIBH	Deep inspiration breath-hold
LAD	Left anterior descending
LVL	Average of mean level
RIHD	Radiation-induced heart disease
RPD	Reproducibility
STB	Stability
SGRT	Surface-guided radiation therapy
VD	Averages of end-to-end deviation
VF	Visual feedback

## References

[1] Cheng Y.J., Nie X.Y., Ji C.C., Lin X.X., Liu L.J., Chen X.M., Yao H., Wu S.H. (2017). Long--term cardiovascular risk after radiotherapy in women with breast cancer. J. Am. Heart Assoc..

[2] Nielsen K.M., Offersen B.V., Nielsen H.M., Vaage-Nilsen M., Yusuf S.W. (2017). Short and long term radiation induced cardiovascular disease in patients with cancer. Clin. Cardiol..

[3] van den Bogaard V.A.B., Spoor D.S., van der Schaaf A., van Dijk L.V., Schuit E., Sijtsema N.M., Langendijk J.A., Maduro J.H., Crijns A.P.G. (2021). The importance of radiation dose to the atherosclerotic plaque in the left anterior descending coronary artery for radiation-induced cardiac toxicity of breast cancer patients?. Int. J. Radiat. Oncol. Biol. Phys..

[4] Zureick A.H., Grzywacz V.P., Almahariq M.F., Silverman B.R., Vayntraub A., Chen P.Y., Gustafson G.S., Jawad M.S., Dilworth J.T. (2022). Dose to the left anterior descending artery correlates with cardiac events after irradiation for breast cancer. Int. J. Radiat. Oncol. Biol. Phys..

[5] Darby S.C., Ewertz M., McGale P., Bennet A.M., Blom-Goldman U., Brønnum D., Correa C., Cutter D., Gagliardi G., Gigante B. (2013). Risk of ischemic heart disease in women after radiotherapy for breast cancer. N. Engl. J. Med..

[6] Remouchamps V.M., Letts N., Vicini F.A., Sharpe M.B., Kestin L.L., Chen P.Y., Martinez A.A., Wong J.W. (2003). Initial clinical experience with moderate deep-inspiration breath hold using an active breathing control device in the treatment of patients with left-sided breast cancer using external beam radiation therapy. Int. J. Radiat. Oncol. Biol. Phys..

[7] Vikström J., Hjelstuen M.H., Mjaaland I., Dybvik K.I. (2011). Cardiac and pulmonary dose reduction for tangentially irradiated breast cancer, utilizing deep inspiration breath-hold with audio-visual guidance, without compromising target coverage. Acta Oncol..

[8] Hayden A.J., Rains M., Tiver K. (2012). Deep inspiration breath hold technique reduces heart dose from radiotherapy for left--sided breast cancer. J. Med. Imag. Radiat. Oncol..

[9] Rochet N., Drake J.I., Harrington K., Wolfgang J.A., Napolitano B., Sadek B.T., Shenouda M.N., Keruakous A.R., Niemierko A., Taghian A.G. (2015). Deep inspiration breath-hold technique in left-sided breast cancer radiation therapy: Evaluating cardiac contact distance as a predictor of cardiac exposure for patient selection. Pract. Radiat. Oncol..

[10] Lu Y., Yang D., Zhang X., Teng Y., Yuan W., Zhang Y., He R., Tang F., Pang J., Han B. (2022). Comparison of deep inspiration breath hold versus free breathing in radiotherapy for left sided breast cancer. Front. Oncol..

[11] Falco M., Masojć B., Macała A., Łukowiak M., Woźniak P., Malicki J. (2021). Deep inspiration breath hold reduces the mean heart dose in left breast cancer radiotherapy. Radiol. Oncol..

[12] Wu J., Yang F., Li J., Wang X., Yuan K., Xu L., Wu F., Tang B., Orlandini L.C. (2024). Reproducibility and stability of voluntary deep inspiration breath hold and free breath in breast radiotherapy based on real-time 3-dimensional optical surface imaging system. Radiat. Oncol..

[13] Cerviño L.I., Gupta S., Rose M.A., Yashar C., Jiang S.B. (2009). Using surface imaging and visual coaching to improve the reproducibility and stability of deep-inspiration breath hold for left-breast-cancer radiotherapy. Phys. Med. Biol..

[14] Hoshina M., Noguchi M., Sekihara H., Masuda K., Shinmura M., Sugahara S. (2024). Chest wall to heart distance reproducibility in postoperative deep inspiration breath-hold radiotherapy for left-sided breast cancer using an Anzai laser sensor with visual feedback. Cureus.

[15] Penninkhof J., Fremeijer K., Offereins-van Harten K., van Wanrooij C., Quint S., Kunnen B., Hoffmans-Holtzer N., Swaak A., Baaijens M., Dirkx M. (2022). Evaluation of image-guided and surface-guided radiotherapy for breast cancer patients treated in deep inspiration breath-hold: A single institution experience. Tech. Innov. Patient Support Radiat. Oncol..

[16] Yamauchi R., Mizuno N., Itazawa T., Masuda T., Akiyama S., Kawamori J. (2021). Assessment of visual feedback system for reproducibility of voluntary deep inspiration breath hold in left-sided breast radiotherapy. J. Med. Imaging Radiat. Sci..

[17] Yoshitake T., Nakamura K., Shioyama Y., Nomoto S., Ohga S., Toba T., Shiinoki T., Anai S., Terashima H., Kishimoto J. (2008). Breath-hold monitoring and visual feedback for radiotherapy using a charge-coupled device camera and a head-mounted display: System development and feasibility. Radiat. Med..

[18] George R., Chung T.D., Vedam S.S., Ramakrishnan V., Mohan R., Weiss E., Keall P.J. (2006). Audio-visual biofeedback for respiratory-gated radiotherapy: Impact of audio instruction and audio-visual biofeedback on respiratory-gated radiotherapy. Int. J. Radiat. Oncol. Biol. Phys..

[19] Goossens S., Senny F., Lee J.A., Janssens G., Geets X. (2014). Assessment of tumor motion reproducibility with audio--visual coaching through successive 4D CT sessions. J. Appl. Clin. Med. Phys..

[20] Linthout N., Bral S., Van de Vondel I., Verellen D., Tournel K., Gevaert T., Duchateau M., Reynders T., Storme G. (2009). Treatment delivery time optimization of respiratory gated radiation therapy by application of audio-visual feedback. Radiother. Oncol..

[21] Nangia S., Khosa R., Piyushi D., Singh M., Singh G., Sreedevi K., Chauhan S.K., Rout S.K., Oomen S. (2023). Deep inspiratory breath-hold radiation for left-sided breast cancer using novel frame-based tactile feedback. J. Med. Phys..

[22] Sano N., Saito M., Onishi H., Kuriyama K., Komiyama T., Marino K., Aoki S., Araya M. (2018). Audio-visual biofeedback for respiratory motion management: Comparison of the reproducibility of breath-holding between visual and audio guidance. J. Mod. Phys..

[23] Yu J., Choi J.H., Ma S.Y., Jeung T.S., Lim S. (2015). Comparison between audio-only and audiovisual biofeedback for regulating patients’ respiration during four-dimensional radiotherapy. Radiat. Oncol. J..

[24] Kefeli A.U., Diremsizoglu U., Erdogan S., Karabey A.U., Konuk A.O., Tirpanci B., Aksu M.G., Sarper E.B. (2025). Patient coaching for deep inspiration breath hold decreases set-up duration and left anterior descending artery dose for left-sided breast cancer radiotherapy. Support. Care Cancer.

[25] Schönecker S., Angelini L., Gaasch A., Zinn A., Konnerth D., Heinz C., Xiong Y., Unger K., Landry G., Meattini I. (2024). Surface-based deep inspiration breath-hold radiotherapy in left-sided breast cancer: Final results from the SAVE-HEART study. ESMO Open.

