# Supplementary material for: Assessment of the Effectiveness of a Visual Coaching Device Combined with Auditory Instructions on Reproducibility and Stability in Deep Inspiration Breath-Hold Radiotherapy
Source: J Clin Med. 2025 Oct 14;14(20):7259. doi: 10.3390/jcm14207259 (PMC12564756; doi:10.3390/jcm14207259)
Supplement: Supplementary file 1 [file jcm-14-07259-s001.zip › jcm-3892578-supplementary.pdf]

Table S1. Reproducibility and stability results of 20 patients across 400 treatment sessions

Patient	Mean_RPD	Mean_STB	Intra_STB	Mean_LVL	STD_LVL	CV_LVL	Mean_VD	STD_VD	CV_VD
1	0.2345	0.4997	0.6569	9.1527	0.1201	0.0131	0.2904	0.1232	0.4241
2	0.6066	0.6368	0.6860	10.6173	0.2266	0.0213	0.412	0.1379	0.3348
3	1.1202	1.1143	1.1905	13.8846	0.3765	0.0271	0.7607	0.2189	0.2877
4	0.5652	0.6818	0.9281	15.9325	0.1822	0.0114	0.4397	0.1387	0.3154
5	0.1966	0.5073	0.6710	9.8053	0.0746	0.0076	0.3021	0.1302	0.4309
6	0.4859	0.8600	0.7339	22.3595	0.1375	0.0061	0.4826	0.1129	0.2339
7	0.8557	0.6592	0.8702	15.3872	0.2601	0.0169	0.4475	0.1669	0.3729
8	0.4770	0.7785	0.6677	22.6552	0.1904	0.0084	0.4399	0.1237	0.2811
9	0.3967	0.9826	0.9689	12.3667	0.1645	0.0133	0.6566	0.156	0.2375
10	0.7840	1.1612	1.2338	17.6087	0.4002	0.0227	0.8317	0.1569	0.1886
11	0.3760	0.5933	0.5510	9.6489	0.0955	0.0099	0.2752	0.1056	0.3837
12	0.2805	0.5616	0.5724	11.1292	0.2309	0.0208	0.3478	0.0878	0.2523
13	0.4347	0.8434	0.8113	19.3744	0.2037	0.0105	0.4566	0.1371	0.3002
14	0.4822	0.6188	0.5026	21.8674	0.1472	0.0067	0.3355	0.0803	0.2393
15	0.4342	0.9511	0.9742	16.3055	0.2135	0.0131	0.6872	0.1160	0.1688
16	0.3727	0.6863	0.9102	12.7605	0.1702	0.0133	0.4358	0.1729	0.3967
17	0.7244	0.4966	0.5977	13.4972	0.2049	0.0152	0.2671	0.0939	0.3517
18	0.5488	0.9265	0.9892	9.9983	0.1624	0.0162	0.5942	0.1967	0.331
19	0.3444	1.0275	0.9755	9.6481	0.2182	0.0226	0.688	0.1576	0.2291
20	0.4283	0.7439	0.6638	12.6958	0.1649	0.0130	0.4821	0.0991	0.2055
Mean ± SD	0.51 ± 0.22	0.77 ± 0.21	0.81 ± 0.21	14.33 ± 4.46	0.20 ± 0.08	0.01 ± 0.01	0.48 ± 0.17	0.14 ± 0.04	0.30 ± 0.08

Abbreviations: RPD, reproducibility; STB, stability; LVL, average of mean level; VD, averages of end-to-end deviation; Mean_RPD, averaging RPD; Mean_LVL, averaging LVL; Mean_STB, averaging STB; Mean_VD, averaging VD; STD_LVL, standard deviations of LVL across all fractions; STD_VD, standard deviations of VD across all fractions; CV_LVL, coefficients of variation of LVL; CV_VD, coefficients of variation of VD; Intra_STB, intra-patient stability representing patient's overall breath-hold performance for the entire therapeutic period.

Table S2. Mean isocenter shift with daily image-guided radiation therapy

**Parameter**	**Mean ± SD**
Vrt (cm)	-0.23 ± 0.23
Lng (cm)	0.06 ± 0.37
Lat (cm)	-0.03 ± 0.15
Pitch (°)	0.15 ± 1.08
Roll (°)	0.05 ± 0.56
Rtn (°)	0.00 ± 0.77

Abbreviations: Vrt, vertical; Lng, long; Lat, lateral; Rtn, rotation; SD, standard deviation.

Table S3. Representative studies on DIBH using visual and/or auditory guidance

Author (Year)	Population / Site (n)	Guidance modality	Measurement method	Key outcomes
Cerviño et al., 2009 [13]	Left-sided breast cancer (n = 5)	Visual feedback (video goggle)	3D surface imaging (Align RT)	Improved reproducibility and stability of chest wall position with VF
Hoshina et al., 2024 [14]	Left-sided breast cancer (n = 10)	Visual feedback (laser sensor)	Chest wall–heart distance on planning CT	Smaller IQR of chest wall–heart distance with VF
Penninkhof et al., 2022 [15]	Left-sided breast cancer (n = 10)	Visual feedback (Align RT)	Residual error in ventral-dorsal direction	Mean absolute differences smaller with VF
Yamauchi et al., 2021 [16]	Left-sided breast cancer (n = 43)	Visual vs. auditory feedback	Chest wall displacement (fluoroscopy)	VF significantly reduced displacement variability
Yoshitake et al., 2008 [17]	Healthy volunteers (n = 5)	Visual feedback (CCD camera + HMD)	External surrogate with CCD	Demonstrated feasibility
Nangia et al., 2023 [21]	Left-sided breast cancer (n = 10)	Tactile feedback (frame-based) with visual cue	Weekly CBCT images reviewed to assess interfraction reproducibility	Reproducibility ≤ 3 mm in all measurements in 6 of 10 patients and ≤ 5 mm in 8 of 10 patients
Sano et al., 2018 [22]	Thoracic and abdominal tumors (n = 20)	Visual vs. auditory feedback	Distance between an anatomical landmark and tumor	VF and AF improved reproducibility, with no significant differences between the two methods. patients reported preference for AF.
Yu et al., 2015 [23]	Healthy volunteers (n = 6)	Audio-only vs. audiovisual	Comparison between guiding and respiration curves (ReMM system)	Regularity between audiovisual and audio-only was similar
Jo et al., 2025 (present study)	Early-stage left breast cancer (n = 20; 400 sessions)	Combined audiovisual feedback	Real-time respiratory signals were recorded to derive nine metrics, including reproducibility and stability	Mean RPD = 0.51 mm; mean STB = 0.77 mm; progressive improvement in both metrics across fractions

Abbreviations: 3D, three-dimensional; IQR, interquartile range; VF, visual feedback; AF, auditory feedback; CCD camera, Charge-couple device camera; HMD, head-mounted display; CBCT, cone beam computed tomography; ReMM, respiration monitoring mask; RPD, reproducibility; STB, stability.
